# Mesit­yl(2,4,6-trimeth­oxy­phen­yl)borinic acid

**DOI:** 10.1107/S160053681002297X

**Published:** 2010-06-18

**Authors:** Sergiusz Luliński, Janusz Serwatowski

**Affiliations:** a Physical Chemistry Department, Faculty of Chemistry, Warsaw University of Technology, Noakowskiego 3, 00-664 Warsaw, Poland

## Abstract

In the title mol­ecule, C_18_H_23_BO_4_, the dihedral angle between the least-squares planes of the aromatic rings is 84.88 (3)°. The B atom deviates by 0.202 (1) Å from the least-squares plane of the mesityl ring. All of the meth­oxy groups are approximately coplanar with the 2,4,6-trimeth­oxy­phenyl ring, whereas the BOH group is twisted with respect to it by 19.5°. The borinic OH group is engaged in an intra­molecular O—H⋯O hydrogen bond with one of *ortho*-meth­oxy groups. The mol­ecular structure is stabilized by weak C—H⋯O contacts. In the crystal, mol­ecules are linked by weak C—H⋯O and C—H⋯π inter­actions, generating a three-dimensional network.

## Related literature

For background to *ortho*-alk­oxy­aryl­boronic acids, see: (Dąbrowski *et al.* 2008 ▶; Luliński (2008 ▶). For related structures, see: Beringhelli *et al.* (2003 ▶); Cornet *et al.* (2003 ▶); Entwistle *et al.* (2007 ▶); Kuhlmann *et al.* (2008 ▶); Weese *et al.* (1987 ▶).
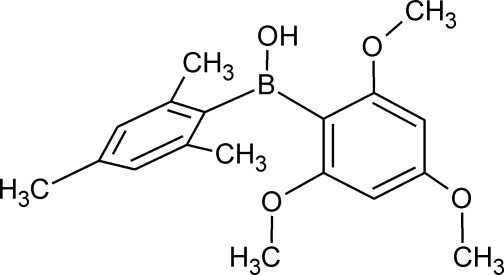


## Experimental

### 

#### Crystal data

C_18_H_23_BO_4_
*M* *_r_* = 314.17
Monoclinic, 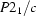

*a* = 6.7775 (2) Å
*b* = 13.0005 (4) Å
*c* = 19.6234 (7) Å
β = 98.895 (3)°
*V* = 1708.24 (10) Å^3^
*Z* = 4
Mo *K*α radiation
μ = 0.08 mm^−1^
*T* = 100 K
0.61 × 0.40 × 0.17 mm

#### Data collection

Oxford Diffraction KM-4-CCD diffractometer
Absorption correction: multi-scan (*CrysAlis RED*; Oxford Diffraction, 2005 ▶) *T*_min_ = 0.93, *T*_max_ = 0.99
28861 measured reflections
4194 independent reflections
3326 reflections with *I* > 2σ(*I*)
*R*_int_ = 0.016

#### Refinement

*R*[*F* ^2^ > 2σ(*F* ^2^)] = 0.038
*wR*(*F* ^2^) = 0.119
*S* = 1.15
4194 reflections
216 parameters
H-atom parameters constrained
Δρ_max_ = 0.37 e Å^−3^
Δρ_min_ = −0.25 e Å^−3^

### 

Data collection: *CrysAlis CCD* (Oxford Diffraction, 2005 ▶); cell refinement: *CrysAlis RED* (Oxford Diffraction, 2005 ▶); data reduction: *CrysAlis RED*; program(s) used to solve structure: *SHELXS97* (Sheldrick, 2008 ▶); program(s) used to refine structure: *SHELXL97* (Sheldrick, 2008 ▶); molecular graphics: *DIAMOND* (Brandenburg, 1999 ▶); software used to prepare material for publication: *SHELXL97*.

## Supplementary Material

Crystal structure: contains datablocks I, global. DOI: 10.1107/S160053681002297X/pv2296sup1.cif

Structure factors: contains datablocks I. DOI: 10.1107/S160053681002297X/pv2296Isup2.hkl

Additional supplementary materials:  crystallographic information; 3D view; checkCIF report

## Figures and Tables

**Table 1:** Hydrogen-bond geometry (Å, °) *Cg* is the centroid of the C15–C20 ring.

*D*—H⋯*A*	*D*—H	H⋯*A*	*D*⋯*A*	*D*—H⋯*A*
O2—H2⋯O9	0.84	1.92	2.6262 (11)	141
C21—H21*A*⋯O13	0.98	2.79	3.4825 (15)	128
C10—H10*B*⋯O2^i^	0.98	2.61	3.4920 (14)	149
C12—H12*A*⋯O11^ii^	0.98	2.79	3.5502 (15)	135
C12—H12*C*⋯O2^i^	0.98	2.84	3.6116 (17)	136
C21—H21*C*⋯O2^iii^	0.98	2.82	3.5746 (16)	134
C21—H21*A*⋯O9^iii^	0.98	2.85	3.6086 (15)	135
C10—H10*A*⋯*Cg*^iv^	0.98	2.79	3.3266 (14)	115
C14—H14*A*⋯*Cg*^v^	0.98	2.88	3.5988 (12)	130

Symmetry codes: (i) 
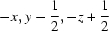
; (ii) 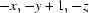; (iii) 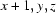; (iv) 
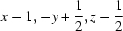
; (v) 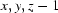.

**Fig. 1.**
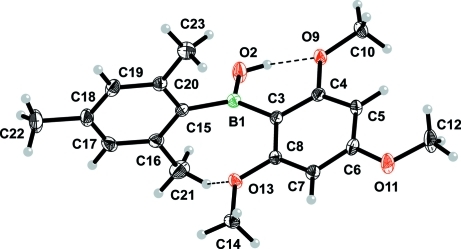
The molecular structure of (I) showing the atom-labelling scheme. The intramolecular hydrogen bond is shown as a dashed lines. Displacement ellipsoids are drawn at the 50% probability level.

**Fig. 2.**
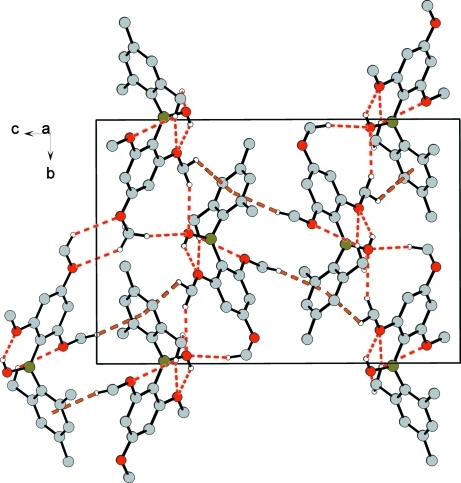
The crystal packing diagram for (I) showing hydrogen-bonding and CH-π interactions (dashed lines).

## supplementary crystallographic information

### Comment

The ability of arylboronic acids to form supramolecular assemblies due to intermolecular hydrogen bonding is well known. The interest in our group has focused on *ortho*-alkoxyarylboronic acids (Dąbrowski *et al.*, 2008; Luliński, 2008), which are related to the title compound, (I). It represents the first structural characterization of borinic acid possessing two different aryl rings. The molecular structure of (I) reflects the significant steric hindrance around the boron atom. The angle C3—B1—C15 is 126.90 (9)°, whereas the dihedral angle between the least squares planes of aryl groups is 84.88 (3)°. The boron atom is deviated from the least squares plane of the mesityl ring by 0.202 (1) Å. The borinic OH group is engaged in an intramolecular O—H···O hydrogen-bond with one of *ortho*-OMe groups. All OMe groups are approximately coplanar with the 2,4,6-trimethoxyphenyl ring, whereas the BOH group is significantly twisted with respect to it. Molecules are linked by C—H···O contacts, of which there are several types (Table 1). Finally, C—H···π interactions occur between *ortho*-OMe groups and the mesityl ring: the distances of H10A and H14A from the ring centroid are 2.79 Å  and 2.88 Å, respectively (Tab. 1). As a result, a three-dimensional network is formed.

The crystal structures of several related arylborinic acids have been reported (Beringhelli *et al.*, 2003; Cornet *et al.*, 2003; Entwistle *et al.*, 2007; Kuhlmann *et al.*, 2008; Weese *et al.*, 1987).

### Experimental

A solution of 2-bromomesitylene (4.0 g) in THF (30 ml) was treated with n-BuLi (2.0 *M* solution in hexanes, 10 ml) at 198 K. The mixture was stirred for 15 min followed by the addition of (2,4,6-trimethoxyphenyl)diethoxyborane (5.30 g). The mixture was quenched with HCl (2.0 *M* solution in diethyl ether), 10 ml). The resulting suspension was filtered. Evaporation yielded an oil which was dissolved in hexane (30 ml). The solution was washed with water (5 ml). The solvent was removed and the residue was triturated with hexane (10 ml). The product was filtered and washed with hexane (10 ml). Crystals suitable for single-crystal X-ray diffraction analysis were grown by slow evaporation of a solution of (I) (0.2 g) in toluene (5 ml).

### Refinement

All hydrogen atoms were located geometrically with C—H  =  0.95 and 0.98 Å for aryl and methyl type H-atoms, respectively, and O—H  = 0.84 Å, and were included in the refinement in the riding model approximation with U_iso_(H) set to 1.2—1.5U_eq_(C/O).

### Crystal data

**Table:**

C_18_H_23_BO_4_	*F*(000) = 672
*M**_r_* = 314.17	*D*_x_ = 1.222 Mg m^−^^3^
Monoclinic, *P*2_1_/*c*	Melting point: 411 K
Hall symbol: -P 2ybc	Mo *K*α radiation, λ = 0.71073 Å
*a* = 6.7775 (2) Å	Cell parameters from 19650 reflections
*b* = 13.0005 (4) Å	θ = 2.2–28.9°
*c* = 19.6234 (7) Å	µ = 0.08 mm^−^^1^
β = 98.895 (3)°	*T* = 100 K
*V* = 1708.24 (10) Å^3^	Prismatic, colourless
*Z* = 4	0.61 × 0.40 × 0.17 mm

### Data collection

**Table:**

Oxford Diffraction KM-4-CCD diffractometer	4194 independent reflections
Radiation source: fine-focus sealed tube	3326 reflections with *I* > 2σ(*I*)
graphite	*R*_int_ = 0.016
Detector resolution: 8.6479 pixels mm^-1^	θ_max_ = 28.6°, θ_min_ = 3.0°
ω scan	*h* = −8→9
Absorption correction: multi-scan (*CrysAlis RED*; Oxford Diffraction, 2005)	*k* = −17→17
*T*_min_ = 0.93, *T*_max_ = 0.99	*l* = −26→26
28861 measured reflections	

### Refinement

**Table:**

Refinement on *F*^2^	Secondary atom site location: difference Fourier map
Least-squares matrix: full	Hydrogen site location: inferred from neighbouring sites
*R*[*F*^2^ > 2σ(*F*^2^)] = 0.038	H-atom parameters constrained
*wR*(*F*^2^) = 0.119	*w* = 1/[σ^2^(*F*_o_^2^) + (0.0686*P*)^2^ + 0.1911*P*] where *P* = (*F*_o_^2^ + 2*F*_c_^2^)/3
*S* = 1.15	(Δ/σ)_max_ = 0.004
4194 reflections	Δρ_max_ = 0.37 e Å^−^^3^
216 parameters	Δρ_min_ = −0.25 e Å^−^^3^
0 restraints	Extinction correction: *SHELXL97* (Sheldrick, 2008), Fc^*^=kFc[1+0.001xFc^2^λ^3^/sin(2θ)]^-1/4^
Primary atom site location: structure-invariant direct methods	Extinction coefficient: 0.0075 (17)

### Special details

**Table:**

Experimental. Yield of (I) 3.5 g, m.p. 410 K. ^1^H NMR (CDCl_3_): 8.62 (br, 1 H), 6.75 (s, 2 H), 6.10 (s, 1 H), 3.85 (s, 3 H), 3.64 (s, 6 H), 2.28 (s, 3 H), 2.22 (s, 6 H) p.p.m.; ^13^C NMR: 167.8, 164.5, 137.4, 135.5, 126.3, 90.8, 55.6, 55.2, 21.7, 21.1 p.p.m.; ^11^B NMR: 52.0 p.p.m..
Geometry. All e.s.d.'s (except the e.s.d. in the dihedral angle between two l.s. planes) are estimated using the full covariance matrix. The cell e.s.d.'s are taken into account individually in the estimation of e.s.d.'s in distances, angles and torsion angles; correlations between e.s.d.'s in cell parameters are only used when they are defined by crystal symmetry. An approximate (isotropic) treatment of cell e.s.d.'s is used for estimating e.s.d.'s involving l.s. planes.
Refinement. Refinement of *F*^2^ against ALL reflections. The weighted *R*-factor *wR* and goodness of fit *S* are based on *F*^2^, conventional *R*-factors *R* are based on *F*, with *F* set to zero for negative *F*^2^. The threshold expression of *F*^2^ > σ(*F*^2^) is used only for calculating *R*-factors(gt) *etc*. and is not relevant to the choice of reflections for refinement. *R*-factors based on *F*^2^ are statistically about twice as large as those based on *F*, and *R*- factors based on ALL data will be even larger.

### Fractional atomic coordinates and isotropic or equivalent isotropic displacement parameters (Å^2^)

**Table:**

	*x*	*y*	*z*	*U*_iso_*/*U*_eq_	
B1	0.23386 (18)	1.01129 (9)	0.18572 (6)	0.0181 (3)	
O2	0.16799 (14)	1.03300 (6)	0.24658 (4)	0.0299 (2)	
H2	0.1060	0.9820	0.2589	0.045*	
C3	0.20989 (15)	0.89979 (8)	0.15476 (5)	0.0162 (2)	
C4	0.06690 (16)	0.83008 (8)	0.17250 (5)	0.0172 (2)	
C5	0.03526 (16)	0.73224 (8)	0.14369 (5)	0.0184 (2)	
H5	−0.0666	0.6886	0.1556	0.022*	
C6	0.15707 (17)	0.70085 (8)	0.09724 (5)	0.0188 (2)	
C7	0.30614 (16)	0.76388 (8)	0.07894 (5)	0.0183 (2)	
H7	0.3905	0.7404	0.0478	0.022*	
C8	0.32920 (15)	0.86210 (8)	0.10719 (5)	0.0159 (2)	
O9	−0.04374 (12)	0.86431 (6)	0.22104 (4)	0.0250 (2)	
C10	−0.20118 (18)	0.80035 (9)	0.23876 (7)	0.0263 (3)	
H10A	−0.2640	0.8343	0.2745	0.039*	
H10B	−0.1457	0.7341	0.2561	0.039*	
H10C	−0.3012	0.7891	0.1977	0.039*	
O11	0.14219 (13)	0.60596 (6)	0.06627 (4)	0.0269 (2)	
C12	0.0047 (2)	0.53478 (10)	0.08829 (7)	0.0334 (3)	
H12A	0.0124	0.4692	0.0643	0.050*	
H12B	−0.1311	0.5624	0.0776	0.050*	
H12C	0.0384	0.5239	0.1382	0.050*	
C14	0.59619 (17)	0.89498 (9)	0.04312 (6)	0.0228 (3)	
H14A	0.6917	0.9494	0.0369	0.034*	
H14B	0.5125	0.8802	−0.0011	0.034*	
H14C	0.6689	0.8327	0.0602	0.034*	
O13	0.47269 (11)	0.92799 (6)	0.09198 (4)	0.02081 (19)	
C15	0.31889 (16)	1.10878 (8)	0.15189 (5)	0.0163 (2)	
C16	0.50993 (16)	1.14768 (9)	0.17717 (6)	0.0208 (2)	
C17	0.57168 (17)	1.24091 (9)	0.15249 (6)	0.0258 (3)	
H17	0.7012	1.2663	0.1699	0.031*	
C18	0.44838 (19)	1.29785 (9)	0.10304 (6)	0.0262 (3)	
C19	0.26153 (18)	1.25828 (9)	0.07763 (6)	0.0236 (3)	
H19	0.1761	1.2957	0.0434	0.028*	
C20	0.19551 (16)	1.16476 (8)	0.10112 (5)	0.0185 (2)	
C21	0.64605 (19)	1.08778 (10)	0.23081 (7)	0.0335 (3)	
H21A	0.6503	1.0158	0.2163	0.050*	
H21B	0.5957	1.0915	0.2749	0.050*	
H21C	0.7808	1.1171	0.2361	0.050*	
C22	0.5173 (2)	1.40011 (10)	0.07841 (8)	0.0414 (4)	
H22A	0.5547	1.4457	0.1181	0.062*	
H22B	0.4087	1.4316	0.0464	0.062*	
H22C	0.6329	1.3893	0.0549	0.062*	
C23	−0.00965 (18)	1.12497 (10)	0.07206 (6)	0.0274 (3)	
H23A	−0.0908	1.1814	0.0496	0.041*	
H23B	−0.0737	1.0969	0.1095	0.041*	
H23C	0.0024	1.0708	0.0382	0.041*	

### Atomic displacement parameters (Å^2^)

**Table:**

	*U*^11^	*U*^22^	*U*^33^	*U*^12^	*U*^13^	*U*^23^
B1	0.0186 (6)	0.0170 (6)	0.0192 (6)	0.0006 (4)	0.0042 (4)	−0.0005 (5)
O2	0.0484 (6)	0.0182 (4)	0.0279 (5)	−0.0070 (4)	0.0214 (4)	−0.0055 (3)
C3	0.0181 (5)	0.0140 (5)	0.0167 (5)	0.0005 (4)	0.0034 (4)	0.0006 (4)
C4	0.0193 (5)	0.0158 (5)	0.0173 (5)	0.0027 (4)	0.0053 (4)	0.0020 (4)
C5	0.0206 (5)	0.0146 (5)	0.0204 (5)	−0.0024 (4)	0.0043 (4)	0.0027 (4)
C6	0.0255 (6)	0.0128 (5)	0.0172 (5)	−0.0004 (4)	0.0009 (4)	−0.0009 (4)
C7	0.0226 (5)	0.0169 (5)	0.0160 (5)	0.0007 (4)	0.0050 (4)	−0.0011 (4)
C8	0.0170 (5)	0.0154 (5)	0.0154 (5)	−0.0006 (4)	0.0024 (4)	0.0013 (4)
O9	0.0306 (5)	0.0161 (4)	0.0332 (5)	−0.0025 (3)	0.0208 (4)	−0.0010 (3)
C10	0.0287 (6)	0.0222 (6)	0.0319 (6)	−0.0031 (5)	0.0169 (5)	0.0047 (5)
O11	0.0402 (5)	0.0156 (4)	0.0275 (4)	−0.0085 (3)	0.0131 (4)	−0.0071 (3)
C12	0.0508 (8)	0.0184 (6)	0.0341 (7)	−0.0141 (5)	0.0166 (6)	−0.0074 (5)
C14	0.0221 (6)	0.0228 (6)	0.0262 (6)	−0.0004 (4)	0.0122 (5)	−0.0025 (5)
O13	0.0226 (4)	0.0178 (4)	0.0248 (4)	−0.0045 (3)	0.0124 (3)	−0.0048 (3)
C15	0.0184 (5)	0.0136 (5)	0.0179 (5)	0.0002 (4)	0.0061 (4)	−0.0043 (4)
C16	0.0189 (5)	0.0182 (5)	0.0257 (5)	0.0000 (4)	0.0055 (4)	−0.0069 (4)
C17	0.0207 (6)	0.0213 (6)	0.0377 (7)	−0.0067 (4)	0.0118 (5)	−0.0114 (5)
C18	0.0361 (7)	0.0157 (5)	0.0314 (6)	−0.0036 (5)	0.0199 (5)	−0.0033 (5)
C19	0.0339 (6)	0.0176 (5)	0.0212 (5)	0.0032 (5)	0.0107 (5)	0.0014 (4)
C20	0.0219 (5)	0.0171 (5)	0.0176 (5)	0.0008 (4)	0.0066 (4)	−0.0023 (4)
C21	0.0249 (6)	0.0304 (7)	0.0413 (7)	0.0023 (5)	−0.0073 (5)	−0.0058 (6)
C22	0.0577 (9)	0.0208 (6)	0.0523 (9)	−0.0102 (6)	0.0295 (7)	−0.0005 (6)
C23	0.0244 (6)	0.0293 (6)	0.0266 (6)	−0.0003 (5)	−0.0022 (5)	0.0013 (5)

### Geometric parameters (Å, °)

**Table:**

B1—O2	1.3675 (14)	C14—H14A	0.9800
B1—C3	1.5704 (15)	C14—H14B	0.9800
B1—C15	1.5803 (16)	C14—H14C	0.9800
O2—H2	0.8400	C15—C20	1.4014 (15)
C3—C4	1.4093 (14)	C15—C16	1.4070 (15)
C3—C8	1.4138 (14)	C16—C17	1.3933 (17)
C4—O9	1.3742 (13)	C16—C21	1.5051 (17)
C4—C5	1.3950 (15)	C17—C18	1.3919 (18)
C5—C6	1.3827 (15)	C17—H17	0.9500
C5—H5	0.9500	C18—C19	1.3863 (17)
C6—O11	1.3719 (13)	C18—C22	1.5128 (17)
C6—C7	1.3904 (15)	C19—C20	1.3981 (16)
C7—C8	1.3911 (15)	C19—H19	0.9500
C7—H7	0.9500	C20—C23	1.5103 (16)
C8—O13	1.3633 (12)	C21—H21A	0.9800
O9—C10	1.4372 (13)	C21—H21B	0.9800
C10—H10A	0.9800	C21—H21C	0.9800
C10—H10B	0.9800	C22—H22A	0.9800
C10—H10C	0.9800	C22—H22B	0.9800
O11—C12	1.4268 (14)	C22—H22C	0.9800
C12—H12A	0.9800	C23—H23A	0.9800
C12—H12B	0.9800	C23—H23B	0.9800
C12—H12C	0.9800	C23—H23C	0.9800
C14—O13	1.4325 (13)		
			
O2—B1—C3	120.01 (10)	H14A—C14—H14C	109.5
O2—B1—C15	113.00 (9)	H14B—C14—H14C	109.5
C3—B1—C15	126.90 (9)	C8—O13—C14	117.95 (8)
B1—O2—H2	109.5	C20—C15—C16	118.65 (10)
C4—C3—C8	115.19 (9)	C20—C15—B1	119.92 (9)
C4—C3—B1	122.13 (9)	C16—C15—B1	121.04 (10)
C8—C3—B1	122.68 (9)	C17—C16—C15	119.98 (11)
O9—C4—C5	120.66 (9)	C17—C16—C21	120.57 (11)
O9—C4—C3	115.65 (9)	C15—C16—C21	119.45 (10)
C5—C4—C3	123.69 (9)	C18—C17—C16	121.65 (11)
C6—C5—C4	117.79 (10)	C18—C17—H17	119.2
C6—C5—H5	121.1	C16—C17—H17	119.2
C4—C5—H5	121.1	C19—C18—C17	118.03 (11)
O11—C6—C5	123.15 (10)	C19—C18—C22	121.41 (12)
O11—C6—C7	114.97 (10)	C17—C18—C22	120.56 (12)
C5—C6—C7	121.87 (10)	C18—C19—C20	121.69 (11)
C6—C7—C8	118.65 (10)	C18—C19—H19	119.2
C6—C7—H7	120.7	C20—C19—H19	119.2
C8—C7—H7	120.7	C19—C20—C15	119.98 (10)
O13—C8—C7	121.98 (9)	C19—C20—C23	119.75 (10)
O13—C8—C3	115.27 (9)	C15—C20—C23	120.26 (10)
C7—C8—C3	122.73 (9)	C16—C21—H21A	109.5
C4—O9—C10	119.04 (9)	C16—C21—H21B	109.5
O9—C10—H10A	109.5	H21A—C21—H21B	109.5
O9—C10—H10B	109.5	C16—C21—H21C	109.5
H10A—C10—H10B	109.5	H21A—C21—H21C	109.5
O9—C10—H10C	109.5	H21B—C21—H21C	109.5
H10A—C10—H10C	109.5	C18—C22—H22A	109.5
H10B—C10—H10C	109.5	C18—C22—H22B	109.5
C6—O11—C12	117.12 (9)	H22A—C22—H22B	109.5
O11—C12—H12A	109.5	C18—C22—H22C	109.5
O11—C12—H12B	109.5	H22A—C22—H22C	109.5
H12A—C12—H12B	109.5	H22B—C22—H22C	109.5
O11—C12—H12C	109.5	C20—C23—H23A	109.5
H12A—C12—H12C	109.5	C20—C23—H23B	109.5
H12B—C12—H12C	109.5	H23A—C23—H23B	109.5
O13—C14—H14A	109.5	C20—C23—H23C	109.5
O13—C14—H14B	109.5	H23A—C23—H23C	109.5
H14A—C14—H14B	109.5	H23B—C23—H23C	109.5
O13—C14—H14C	109.5		
			
O2—B1—C3—C4	22.19 (16)	C7—C6—O11—C12	174.12 (10)
C15—B1—C3—C4	−154.17 (11)	C7—C8—O13—C14	2.63 (15)
O2—B1—C3—C8	−157.91 (10)	C3—C8—O13—C14	−178.89 (9)
C15—B1—C3—C8	25.73 (17)	O2—B1—C15—C20	−95.70 (12)
C8—C3—C4—O9	177.19 (9)	C3—B1—C15—C20	80.87 (14)
B1—C3—C4—O9	−2.90 (15)	O2—B1—C15—C16	77.06 (13)
C8—C3—C4—C5	−2.93 (15)	C3—B1—C15—C16	−106.37 (13)
B1—C3—C4—C5	176.97 (10)	C20—C15—C16—C17	1.03 (15)
O9—C4—C5—C6	−177.41 (10)	B1—C15—C16—C17	−171.82 (10)
C3—C4—C5—C6	2.72 (16)	C20—C15—C16—C21	−178.94 (10)
C4—C5—C6—O11	179.07 (10)	B1—C15—C16—C21	8.21 (16)
C4—C5—C6—C7	−0.35 (16)	C15—C16—C17—C18	0.20 (17)
O11—C6—C7—C8	178.98 (9)	C21—C16—C17—C18	−179.83 (11)
C5—C6—C7—C8	−1.55 (16)	C16—C17—C18—C19	−1.15 (17)
C6—C7—C8—O13	179.63 (9)	C16—C17—C18—C22	178.50 (11)
C6—C7—C8—C3	1.26 (16)	C17—C18—C19—C20	0.87 (17)
C4—C3—C8—O13	−177.59 (9)	C22—C18—C19—C20	−178.77 (11)
B1—C3—C8—O13	2.50 (15)	C18—C19—C20—C15	0.36 (16)
C4—C3—C8—C7	0.88 (15)	C18—C19—C20—C23	179.78 (10)
B1—C3—C8—C7	−179.03 (10)	C16—C15—C20—C19	−1.30 (15)
C5—C4—O9—C10	−3.75 (15)	B1—C15—C20—C19	171.63 (10)
C3—C4—O9—C10	176.13 (10)	C16—C15—C20—C23	179.28 (10)
C5—C6—O11—C12	−5.34 (16)	B1—C15—C20—C23	−7.79 (15)

### Hydrogen-bond geometry (Å, °)

**Table:**

Cg is the centroid of the C15–C20 ring.

**Table:**

*D*—H···*A*	*D*—H	H···*A*	*D*···*A*	*D*—H···*A*
O2—H2···O9	0.84	1.92	2.6262 (11)	141
C21—H21A···O13	0.98	2.79	3.4825 (15)	128
C10—H10B···O2^i^	0.98	2.61	3.4920 (14)	149
C12—H12A···O11^ii^	0.98	2.79	3.5502 (15)	135
C12—H12C···O2^i^	0.98	2.84	3.6116 (17)	136
C21—H21C···O2^iii^	0.98	2.82	3.5746 (16)	134
C21—H21A···O9^iii^	0.98	2.85	3.6086 (15)	135
C10—H10A···Cg^iv^	0.98	2.79	3.3266 (14)	115
C14—H14A···Cg^v^	0.98	2.88	3.5988 (12)	130

Symmetry codes: (i) −*x*, *y*−1/2, −*z*+1/2; (ii) −*x*, −*y*+1, −*z*; (iii) *x*+1, *y*, *z*; (iv) *x*−1, −*y*+1/2, *z*−1/2; (v) *x*, *y*, *z*−1.
